# Reproductive Rates in Australian Rodents Are Related to Phylogeny

**DOI:** 10.1371/journal.pone.0019199

**Published:** 2011-04-29

**Authors:** Eli Geffen, Kevin C. Rowe, Yoram Yom-Tov

**Affiliations:** 1 Department of Zoology, Tel Aviv University, Tel Aviv, Israel; 2 Museum of Vertebrate Zoology, University of California, Berkeley, California, United States of America; Natural History Museum of Denmark, Denmark

## Abstract

**Background:**

The native rodents of Australia are commonly divided into two groups based on the time of their colonization of the Sahulian continent, which encompasses Australia, New Guinea, and the adjacent islands. The first group, the “old endemics,” is a diverse assemblage of 34 genera that are descended from a single colonization of the continent during the Pliocene. A second group, the “new endemics,” is composed of several native *Rattus* species that are descended from a single colonization during the Pleistocene. Finally, a third group is composed of three non-native species of *Rattus* and *Mus* introduced into Australia by humans over the last 200 years. Previous studies have claimed that the three groups differ in their reproductive rates and that this variation in rates is associated with the unique environmental conditions across Australia. We examined these hypotheses using phylogenetically controlled methods.

**Methodology and Results:**

We examined the relationship between the reproductive rates of the Australian rodents and the environmental variations across the continent, as well as the epoch of their colonization of the continent. Our results revealed no significant correlation with environmental variables but a significant association between colonization age and all the reproductive parameters examined.

**Discussion:**

Based on a larger phylogeny of the subfamily Murinae, we showed that significant differences in reproductive rates among colonization groups are shared with their closest relatives outside Sahul. Therefore, the lower reproductive rates in the old endemics are more likely to be the result of phylogenetic history and conservation of traits than an adaptation to the Australian environment. In the new endemics, we found a trend of increasing reproductive rates with diversification. We suggest that the differences in reproductive rates of the old endemic rodents and the native *Rattus* represent alternative adaptive strategies that have allowed them to utilize similar ecological niches across Australia.

## Introduction

The topography, climate, and soils of Australia make it particularly distinct among the continents of this planet. Australia is the oldest and flattest continent, it has an erratic climate, and its soils are poor in nutrients [1]. Australia is also one of the most isolated continents. Since the break-up of Gondwana in the Cretaceous, Australia has only reached proximity with the Asian continent within the last 10–15 myr, and then only at the very end of the Sunda shelf [2]–[4]. Australia has never been connected by land to the Asian continent, and remains separated by deep ocean channels that demarcate major biogeographic regions [5]–[7]. Southern New Guinea to the north of Australia is part of the same continental shelf, Sahul, while northern New Guinea and the surrounding archipelagos (i.e. the Moluccans) are the result of recent accretions caused by collision with the Asian continental shelf [8].

The birds and mammals that have colonized Australia since its separation from Gondwana are commonly divided into two groups based on the time of their colonization of Sahul [9]. The 64 rodent species native to Australia are all from the subfamily Murinae and are descendants of two colonization events from Southeast Asian rodents (Sahul). The first colonization, which occurred in the Pliocene (4–6 mya; [10]–[11]), resulted in a diverse assemblage of 34 Sahulian genera that are commonly referred to as “old endemics”. The second colonization, in the Pleistocene (1 mya; [12]), referred to here as the “new endemics,” resulted in the several species of native *Rattus* [13]. Finally, in the last two centuries, humans have introduced three non-native species of murine rodents (*R. rattus*, *R. norvegicus* and *Mus musculus*).

The 57 native rodent species of Australia (old endemics), excluding *Rattus*, are part of a larger old endemic radiation of nearly 160 species that evolved from a single colonization event in Sahul [11]. The Australian species of the Sahulian old endemics are the result of several expansions of this group into Australia from New Guinea during the Pliocene and Pleistocene. The old endemics are a morphologically and ecologically diverse group of species. They range in body mass from 10 to 700 g. Their lifestyles include the terrestrial, the semi-aquatic, and the arboreal; and they occur in all of Australia's main habitats, including desert, rainforest, and shrub-land.

The seven species of native Australian *Rattus* (new endemics) are part of a second radiation of 25 species of Sahulian *Rattus* that resulted from a second, more recent colonization of Sahul [12]. Recent phylogenetic analyses supported the monophyly of the native *Rattus* of Sahul and confirmed that the *sordidus* group of rats is highly derived within the Australian *Rattus* [12], [14]–[15]. Six of the Australian new endemics are the result of a single colonization from New Guinea, while the seventh species, *Rattus leucopus*, which is also widespread in New Guinea, may represent a separate colonization [12]. Compared to the old endemics the new endemics are much more conserved morphologically (body mass of 60–130 g) and all are terrestrial in lifestyle. However, like the old endemics, they occupy virtually every habitat and eco-region of Australia.

Finally, the three introduced species include two species of *Rattus* and one species of *Mus*, ranging in body mass from 17 to 350 g. *Rattus norvegicus* is restricted to areas of human habitation and is not commonly found in the native habitats of Australia. *R. rattus* is commensal with humans too but is also found in native rainforest (K.C. Rowe, personal observation) and other coastal forest habitats [16]–[17]. *Mus musculus* is widespread throughout most Australian habitats, including coastal areas and the arid interior [18].

The reproductive rates of Australian birds [19]–[20] and rodents [21] differ between old endemics and new endemics, and in both classes the old endemics produce on average a smaller clutch or litter in comparison with the new endemics. Yom-Tov [21] showed that among the native rodents, the old endemics have longer gestation and weaning periods and achieve sexual maturity later than the new endemics. Compared to most other Southeast Asian murines, the old endemics have smaller litter sizes, longer gestation periods, and fewer nipples; however, until recently the closest relatives of the old endemics were not known [22]. Some studies have suggested that the lower reproductive rates in the old endemic rodents and birds of Australia is an adaptation to the unusual characteristics of the Australian environment, while the higher reproductive rates in the new endemics and introduced species is related to their recent origin on the continent [19]–[21], [23].

Within the native Australian rodents (old endemics and new endemics), reproductive rates are also thought to differ between arid and mesic environments [22] and to be influenced by rainfall variation [24]–[25]. For instance, reproductive rates of species from northern rainforests are generally slower than species from arid or grassland habitats (e.g. *Melomys cervinipes* vs. *Pseudomys desertor* and *R. leucopus* vs. *R. villosissimus*). Considering these environmental factors, primarily land productivity and precipitation, we examined whether reproductive rates differ among colonization stages and habitats of Australian rodents. We used phylogenetically-controlled methods to examine the hypothesis that the old endemic rodents of Australia have a slower reproductive rate than either the native *Rattus* (new endemics) or the recently introduced species; and where relevant we discuss the phylogenetic and environmental reasons for this phenomenon.

## Methods

We compiled data from the literature on the breeding parameters (litter size, number of nipples, gestation period, weaning period, and age at sexual maturity) for all but one of the 64 native Australian rodent species and for each of three non-native species ([18], [22], [26]–[27]; Table S1). Data on mean adult body mass were compiled from [28]. When a range of values was reported, we used the mean value. We categorized the above species into “old endemics”, “new endemics” (i.e., native *Rattus* species), and “introduced species” (*R. rattus*, *R. norvegicus* and *Mus musculus*) based on the epoch in which they had colonized Sahul (Pliocene, Pleistocene, Historical, respectively). We used non-parametric statistics throughout (i.e., Kruskal-Wallis test, Spearman ranked correlation and permutation tests) because the breeding variables (except period to sexual maturity) did not distribute normally, even after applying the Box-Cox transformation. Using the Kruskal-Wallis test, we compared breeding parameters among species belonging to the three colonization ages. Breeding data were controlled for the effect of body mass only in cases where the two variables were significantly correlated.

**Figure 1:**
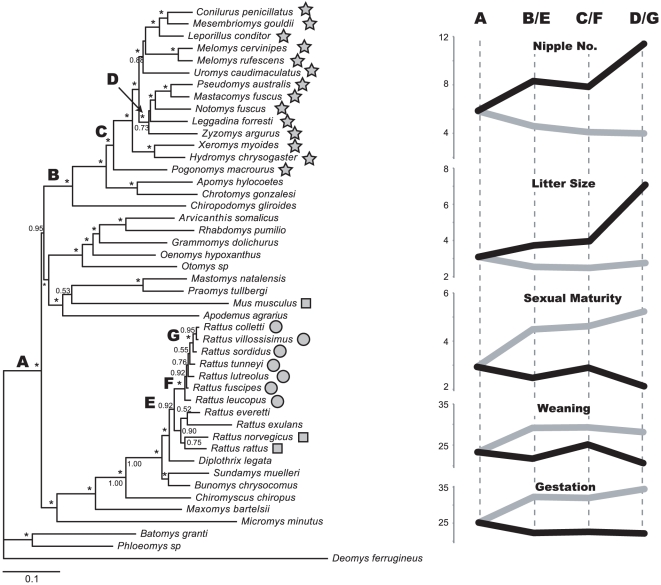
Phylogeny of the Australian rodents and reconstructed ancestry on key nodes in the phylogeny (labeled as nodes A–G). Bayesian posterior probabilities indicated at the nodes. Nodes supported by 1.00 are marked with “*”. Shapes beside terminal taxa designate species in the three Australian colonization stages (star = old endemics, circle = new endemics, and square = introduced species). All other rodent species are non-Australian and are presented for reference. *Melomys rufescens* and *Pogonomys macrourus* were used only for positioning these two genera on the phylogenetic tree, and served as a phylogenetic reference for the Australian species *Melomys cervinipes* and *Pogonomys mollipilosus* for which we had no genetic sequences. Line graphs on the right side of the figure show the values of the ancestral breeding parameter states. They are drawn for both the old endemics (gray line, nodes A, B, C, and D) and the new endemics (black line, nodes A, E, F, and G), starting at node A and increasing toward more recent nodes.

To examine how reproductive rates have evolved following the colonization of Sahul, we reconstructed ancestral states for each reproductive parameter on a molecular phylogeny. Our phylogenetic analyses comprised 45 species from the rodent subfamily Murinae and 1 species from the Deomyinae that was used as an outgroup. These molecular data included 13 species representing all genera of old endemic Australian rodents, all 7 species of new endemics, and all 3 introduced species (*Rattus* and *Mus*). These 24 species represent a subset of the 63 Australian species in our analyses. We estimated our phylogeny based on DNA sequence data from 6 unlinked nuclear autosomal loci (945 bp for GHR, 2710 bp for BRCA1, 3074 bp for RAG1, 1122 bp for BDR, 1316 bp for IRBP, and 435 bp for AP5; [11]–[12]). Phylogenies were estimated using Bayesian methods as described in [12]. We estimated ancestral states of all reproductive variables at seven key nodes in our phylogeny. Node A, near the root of Murinae, represents the most recent common ancestor of all Australian rodents in our analyses (old endemics, new endemics, and introduced, Fig. 1). Node B represents the common ancestor of the old endemics and their nearest relatives outside Sahul. Node C represents the most recent common ancestor of the old endemic rodents of Australia. Node D represents the common ancestor of the Pseudomys division, a derived, monophyletic lineage of old endemics (*Pseudomys*, *Mastacomys*, *Notomys*, *Leggadina*, *Zyzomys*) that evolved within Australia. Node E represents the common ancestor of all *Rattus*, i.e. the nearest relatives of the new endemics outside Sahul. Node F represents the most recent common ancestor of the new endemics. Finally, node G represents the common ancestor of the sordidus species group, which is a derived, monophyletic lineage that evolved within Australia (Table 1, Fig. 1). We reconstructed ancestral states using weighted squared-change parsimony [29] as implemented in the Mesquite software package [30]. This approach is equivalent to a maximum likelihood estimate assuming a Brownian motion model of evolution and is appropriate for continuous characters.

**Table 1:** Ancestral state reconstruction for breeding parameters at seven nodes on the phylogeny of Murinae (see Fig. 1).

	Node A	Node B	Node C	Node D	Node E	Node F	Node G
Body weight	250.46	92.41	130.09	112.63	321.29	195.13	106.88
Litter size	3.10	2.54	2.49	2.76	3.73	3.97	7.08
Nipple number	5.85	4.61	4.10	4.01	8.32	7.86	11.41
Gestation period	25.18	32.26	31.92	34.43	22.31	22.65	22.18
Weaning period	23.32	29.17	29.34	28.20	21.74	25.21	20.71
Sexual maturity	2.94	4.48	4.62	5.23	2.48	2.90	2.14

We also examined whether differences in breeding parameters among and within colonization stages can be explained by habitat and climate variations. To address this issue we calculated five average environmental variables for the regions where the species are found: Net Primary Production (NPP; gC m^2^ yr^−1^), total precipitation, total rain days, coefficient of variation in annual rainfall, and mean annual temperature. These climatic variables were chosen because the data are readily available and because they distinguish among the major habitats of Australia, particularly arid, seasonally wet habitats and the more stable mesic habitats such as rainforest. Averages of environmental variables for each species were obtained by superimposing high-resolution climatic data (one degree latitude and longitude intervals during the entire 20^th^-century; data were kindly provided by Dr. Mark Lomas, University of Sheffield) on each species' historical range digitized from [18]. We used a Principal Component Analysis (PCA) to reduce the number of variables and eliminate co-linearity among them. We then regressed each of the breeding parameters (dependent variable) against the factors retained by the PCA (i.e., eigenvalue ≥1.0) and their interactions. The program JMP (version 8, SAS Inc.) was used for all calculations of PCA and linear regressions.

Climatic variables are often a reliable proxy for eco-regions and habitats. We used our high-resolution climatic data to test for differences among key eco-regions in Australia. Each one-degree latitude and longitude cell was assigned an eco-region based on the map produced by [31], resulting in the following eco-regions: 1. Deserts and xeric shrubland, 2. Mediterranean forest, woodland and scrub, 3. temperate broadleaf and mixed forest, 4. temperate grassland, savanna and shrubland, 5. tropical and subtropical grassland, savanna and shrubland, and 6. tropical and subtropical moist broadleaf forest. Montane grassland and shrubland were omitted because the total land cover of this habitat was negligible. For comparison among eco-regions using the above PCA factors and their interaction we used a non-parametric distance-based MANOVA (NPMANOVA; [32]). NPMANOVA is based on the distance matrix between all pairs of observations (we calculated the Euclidean distance between eco-regions based on the climatic variables). The procedure is nonparametric since the P value is calculated by 10,000 randomizations of the distance matrix. Post-hoc pairwise comparisons were corrected using the Bonferroni method [33]. This procedure was added in order to confirm that the climatic variables we selected can reliably separate between eco-regions in Australia, and thus can reflect not only the association with climate but also with habitat type.

We accounted for the contribution of phylogeny in our comparative analysis using phylogenetic eigenvector regression (PVR; [34]). This approach exploits PCA to extract informative eigenvectors from a phylogenetic distance matrix. We calculated maximum likelihood distance matrix, using DNA sequence from six nuclear loci, to represent phylogenetic differences among taxa. The number of eigenvectors to retain was determined by testing for significant phylogenetic signature using correlograms with increased number of eigenvectors [34]–[35]. Correlograms were based on the Moran's I autocorrelation measure, and calculated using the program PA [35]. Next, the original breeding traits were regressed against the dominant retained eigenvectors. The residuals, resulting from a regression on each of the breeding variables against the selected eigenvectors, are species-specific phylogenetically-free data (correlogram Z≤1.96), suitable for testing for climatic and environmental effects.

## Results

The traditional division of Australian rodents into three distinct colonization stages is supported by consideration of the colonization of broader Sahul. Phylogenetic analyses (Fig. 1) clearly show that the Australian rodents (native and introduced) are derived from three separate lineages within the Murinae (the Sahulian old endemics, *Rattus*, and *Mus*; see also [11]). These three lineages share a common ancestor near the root of the Eumurinae (Murinae, excluding the Philippine cloud rats *Phloeomys* and *Batomys*; [36]) and are not closely related within the Murinae (Fig. 1, Node A). The first two colonization stages (old endemics and new endemics) each reflect a single colonization of Sahul and subsequent radiation, while the last colonization stage (*R. rattus*, *R. norvegicus* and *Mus musculus*) reflects three independent colonizations but all within historical times. As reported previously [11], we recovered a monophyletic lineage of Sahulian old endemic rodents that is a sister clade of Philippine rodents and the Southeast Asian rodent *Chiropodomys* [10] (Fig. 1, Node B). Within the old endemic rodents of Sahul the first split is between *Pogonomys* and all other genera (Fig. 1, Node C). The lineage containing *Pseudomys*, *Mastacomys*, *Notomys*, *Leggadina*, and *Zyzomys* (Fig. 1, Node D) is nested within the Australian old endemics and therefore a derived lineage. The new endemics (native Australian *Rattus*) formed a monophyletic lineage within *Rattus*. They shared a common ancestor with the two introduced *Rattus* species at the root of all *Rattus* [11]–[12], [15], and therefore are not closely related within the genus (Fig. 1, Node E). Within the new endemics (Fig. 1, Node F), the first split was between *R. leucopus* and the other six species [12]. The *sordidus* group (*R. sordidus*, *R. colletti*, *R. villosissimus*) is nested within the Australian new endemics and therefore a derived lineage (Fig. 1, Node G).

The three colonization stages have overlapping ranges of body mass (10–700, 60–130 and 17–350 g for the old endemics, new endemics, and introduced species, respectively) and body mass was not significantly correlated with any of the breeding variables for the complete set of 63 species (Spearman rank correlation; litter size: r_52_ = −0.158, P = 0.263; number of nipples: r_33_ = 0.276, P = 0.120; gestation period: r_40_ = 0.036, P = 0.825; weaning period: r_38_ = 0.022, P = 0.894; sexual maturity: r_36_ = 0.272, P = 0.108). The subset of 24 species used in our phylogenetic analyses also showed no significant correlation between body mass and any of the breeding variables, except for sexual maturity (litter size: r_24_ = −0.151, P = 0.481; number of nipples: r_21_ = 0.094, P = 0.678; gestation period: r_22_ = 0.281, P = 0.205; weaning period: r_21_ = 0.257, P = 0.261; sexual maturity: r_20_ = 0.439, P = 0.046). In light of the above results, we did not control for the effect of body mass in any analysis, except those that involved sexual maturity.

**Figure 2:**
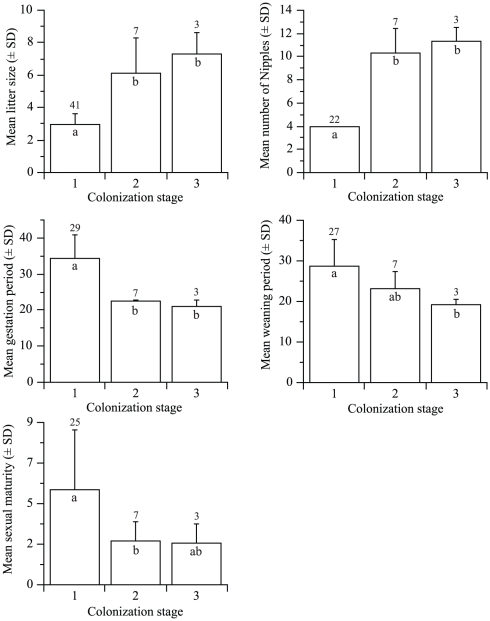
Mean (± SD) litter size, number of nipples, gestation period (days), weaning period (days), and age of sexual maturity (months) for the species of each colonization stage. Values above error bars are sample size. Mean colonization stages not connected by the same letter (inside bar) are significantly different (P<0.05).

We did not detect significant variation in body mass among the three different colonization stages when considering all 63 Australian rodent species (Kruskal-Wallis test; χ^2^_2_ = 4.3, P = 0.120). However, species of the different colonization stages were significantly different for all breeding characters (litter size: χ^2^_2_ = 22.0, P<0.0001; number of nipples: χ^2^_2_ = 29.9, P<0.0001; gestation period: χ^2^_2_ = 22.0, P<0.0001; weaning period: χ^2^_2_ = 8.6, P = 0.013; sexual maturity: χ^2^_2_ = 8.9, P = 0.011). As a general trend, the old endemic species had significantly smaller litter size (multiple comparisons; 1–2: Z = 3.8, P = 0.0004; 1–3: Z = 3.0, P = 0.009), significantly fewer nipples (1–2: Z = 3.8, P = 0.0004; 1–3: Z = 2.9, P = 0.01), and significantly longer gestation period (1–2: Z = 3.8, P = 0.0004; 1–3: Z = 3.2, P = 0.004) compared to the species of the later colonization stages (Fig. 2). Weaning period of the old endemic species was also significantly longer (Z = 2.6, P = 0.029) than in the introduced species, and sexual maturity was reached significantly later (Z = 2.5, P = 0.033) in the old compared to the new endemics (Fig. 2).

**Figure 3:**
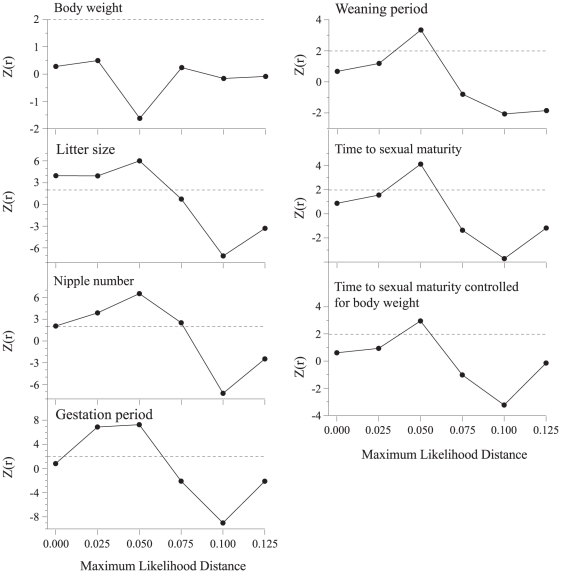
Moran's correlograms for body weight, litter size, number of nipples, gestation period, weaning period, time to sexual maturity, and time to sexual maturity controlled for body weight. The dashed line indicates the significance cut-off value (Z≥1.96).

Our phylogeny showed that the definition of colonization stages corresponds to two distinct native clades and three separate lineages of non-native rodents (Fig. 1). This tree includes all species of the new endemics and at least one representative of every genus of the old endemics. Therefore, this limited phylogeny properly represents the evolutionary relationships and divergence of Australian rodent genera and is representative of phylogenetic correlations in the full data set. We detected similar life-history trends in this phylogenetically defined data set as were shown above for the full set of 63 species. Mean body mass did not differ significantly among colonization stages (Kruskal-Wallis test; χ^2^_2_ = 0.04, P = 0.982), whereas all breeding characters were significantly different among the colonization stages (litter size: χ^2^_2_ = 15.0, P = 0.0006; number of nipples: χ^2^_2_ = 18.0, P = 0.0001; gestation period: χ^2^_2_ = 16.0, P = 0.0003; weaning period: χ^2^_2_ = 7.8, P = 0.021; time to sexual maturity: χ^2^_2_ = 8.0, P = 0.019). Mean age of sexual maturity, controlled for body mass, was also significantly different among colonization stages (χ^2^_2_ = 7.3, P = 0.026). All breeding characters showed a significant phylogenetic autocorrelation at a maximum likelihood distance <0.06–0.08 (Z≥1.96; Fig. 3), except for body mass (max. Z = 0.5). These results imply that all the breeding variables should be controlled for phylogeny, while body mass should not. We tested the performance of the PVR using correlograms. The residuals resulting from the PVR showed no significant phylogenetic autocorrelation in any breeding variable at any level (Z≤1.4 in all correlograms. After removing phylogenetic variation using PVR, the residuals for the breeding parameters were not significantly different among colonization stages for any parameter (Kruskal-Wallis test; litter size: χ^2^_2_ = 1.6, P = 0.461; number of nipples: χ^2^_2_ = 0.7, P = 0.708; gestation period: χ^2^_2_ = 0.6, P = 0.776; weaning period: χ^2^_2_ = 0.5, P = 0.780; sexual maturity: χ^2^_2_ = 0.5, P = 0.793). These results indicate that no significant differences in breeding parameters among colonization stages remained after accounting for phylogenetic variation.

One of the hypotheses of breeding parameters in Australian vertebrates is that they have evolved to be at slower pace with time since colonization. To address this issue we estimated ancestral breeding parameter states at nodes on the phylogeny representing (1) the ancestral state at the root of Murinae, (2) the ancestral state outside Sahul, (3) the ancestral state at the root of the colonization of Australia, and (4) the ancestral state of a derived clade that evolved within Australia after colonization (Fig. 1; Table 1). Since reproductive traits for the introduced species did not evolve in Australia, and because the introduced species do not form a single lineage, we did not include them in these analyses. For the old endemics, the trend is toward a lower reproductive rate relative to the ancestral state at the root of all Murinae. However, the most apparent reduction in reproductive rates occurs from node A (Murinae) to node B, representing the common ancestor of the Australian old endemics and their relatives outside Sahul. Indeed, none of the breeding parameters significantly differ between the Australian old endemics and their closest relatives outside Sahul (Kruskal-Wallis test; χ^2^_1_≤2.6, P≥0.11 for all breeding parameters). From their ancestral state outside Sahul (node B) to the common ancestor of all old endemics (node C), and to the specifically Australian-evolved lineage of the *Pseudomys* division (node D), there is no strong trend in evolution of breeding variables (Fig. 1). The reverse trend is apparent in the new endemics, with increasing reproductive rates from the root of Murinae (Node A) to the common ancestor of all *Rattus* (Node E; Fig. 1). As in the old endemics, there is little apparent change in breeding parameters from the ancestral state outside Sahul (Node E) to the common ancestor of the Australian new endemics (Node F). However, there is a clear increase in reproductive rates in the *sordidus* group (Node G) relative to their common ancestor with the other new endemics (Node F). Litter size and number of nipples were significantly higher (Kruskal-Wallis test; χ^2^_2_ = 7.1, P = 0.028 and χ^2^_2_ = 6.6, P = 0.037, respectively) and weaning period was significantly lower (χ^2^_2_ = 6.7, P = 0.035) in the *sordidus* group compared to the other new endemics, whereas gestation period (χ^2^_2_ = 3.4, P = 0.182), sexual maturity (χ^2^_2_ = 1.6, P = 0.561), and body weight (χ^2^_2_ = 0.8, P = 0.676) were not significantly different.

In our climatic PCA analyses, the five climatic variables collapsed into two principal components that together explained 98.0% of the variance. The first component (PC1) contained all the precipitation-related variables (NPP, total precipitation, total rain days, and rainfall CV) and accounted for 67.5% (eigenvalue = 3.37) of the total variance. The second component (PC2) was composed of mean annual temperature and accounted for 30.5% (eigenvalue = 1.52) of the total variance. Using multiple regression, we examined whether these two principal components or their interactions were meaningful predictors of breeding parameters across the Australian rodents. None of the breeding parameters was significantly correlated with either of the climatic components (litter size: R^2^ = 0.048, F_3,20_ = 0.3, P = 0.799; number of nipples: R^2^ = 0.078, F_3,18_ = 0.5, P = 0.682; gestation period: R^2^ = 0.047, F_3,18_ = 0.3, P = 0.829; weaning period: R^2^ = 0.023, F_3,17_ = 0.1, P = 0.940; sexual maturity: R^2^ = 0.14, F_3,17_ = 0.9, P = 0.456). Body mass too was not significantly correlated with climatic effects (R^2^ = 0.043, F_3,20_ = 0.3, P = 0.827). None of the above breeding parameters was significantly correlated with climatic components after controlling for the effect of phylogeny using PVR (R^2^≤0.257, P≥0.158 in all tests).

We found no correlation between breeding parameters and environmental variables within the old endemics (litter size: R^2^ = 0.163, F_3,9_ = 0.6, P = 0.639; number of nipples: 4 in all species analyzed; gestation period: R^2^ = 0.035, F_3,7_ = 0.1, P = 0.966; weaning period: R^2^ = 0.073, F_3,6_ = 0.2, P = 0.920; sexual maturity: R^2^ = 0.429, F_3,6_ = 1.5, P = 0.306) or within the new endemics (litter size: R^2^ = 0.066, F_3,7_ = 0.2, P = 0.916; number of nipples: R^2^ = 0.367, F_3,7_ = 1.3, P = 0.339; gestation period: R^2^ = 0.354, F_3,7_ = 1.3, P = 0.353; weaning period: R^2^ = 0.096, F_3,7_ = 0.2, P = 0.860; sexual maturity: R^2^ = 0.270, F_3,7_ = 0.9, P = 0.503). Moreover, there was no correlation between the PVR residuals for environmental variables and breeding parameters within the old endemics (R^2^≤0.620, P≥0.101 in all regressions) or within the new endemics (R^2^≤0.349, P≥0.362 in all regressions).

The squared Euclidean distances among climatic variables between eco-regions were significantly larger than expected by random (NPMANOVA, F_5,686_ = 136.4, P<0.0001). All Bonferroni-corrected pair-wise comparisons were significant (P≤0.01). These results imply that our high-resolution climatic data are sufficiently powerful to present an adequate profile of the key eco-regions in Australia. Consequently, by linking breeding parameters and climatic variables, we also tested for the effect of habitat/eco-region.

## Discussion

Clearly, the old endemic rodents of Australia have a slower reproductive rate than either the native *Rattus* (new endemics) or the recently introduced species (Fig. 2). These results are consistent with those of [21]. In addition, none of the breeding parameters was significantly associated with climatic variables when considering all species together or each colonization stage independently. Several hypotheses offer alternative explanations for why this disparity exists.

The first hypothesis is that breeding parameters are associated with environmental differences across Australia, and that the differences in the geographic distributions of species from the colonization stages account for their variation in breeding parameters. Our environmental analyses enabled us to reject this hypothesis. If the hypothesis had been true, then climatic variables would be significantly correlated with breeding parameters across all species, which they are not. The lack of significant correlation between climatic and breeding variables is observed even after controlling for phylogeny. The old endemics are found in virtually every habitat in Australia, with 32% of the species distributed in the arid interior of the country, and a similar proportion observed in the new endemics (29%) and the introduced species (33%). These proportions are not significantly different between the colonization stages (Fisher exact test, new endemics and introduced species combined, n = 67, P = 1.0). These results are consistent with the conclusion that, overall, the different habitats in Australia are equally utilized by the species of the different colonization stages.

The second hypothesis is that the Australian environment is more conducive to a slow reproductive pace, and that the newer endemics have not had as much time to adapt to this environment as the old endemics. This hypothesis has been constructed upon several of Australia's characteristics. This continent is the flattest and driest of all the continents, and has a uniquely high proportion of nutrient-poor soils (reviewed by [1]). In most parts of Australia rainfall is erratic and drought is common [37], seasonality of growth response is very high in many areas [38], and the resulting small increment in food availability characterizes much of inland Australia [39]. Several studies have contended that the above physical conditions have influenced the life history of Australian animals. Hence, small clutch (and litter) size, high incidence of cooperative breeding, helping in the nest, nomadism and 48-hour intervals in egg laying, protracted molt, extended parental care, long breeding seasons, and increased longevity are among the biological characteristics of Australian birds and mammals that have been attributed to environmental conditions [19]–[21], [23], [39]–[42].

While these arguments have been based on correlations between the Australian environment and the breeding rates of the old endemic species, they have not considered the phylogenetic relationships of this group to other rodents outside Australia. The old endemic rodents of Australia are part of at least five or six colonizations from tropical New Guinea [11]. The endemic New Guinean rodents exhibit a similarly slow rate of breeding [43]–[44]. Like all Australian old endemics (for which we have data) they have four nipples and have smaller litters and longer gestation times than the new endemics. Compared to most other murines and to the ancestral state of all Murinae (Node A), the old endemics have smaller litter sizes, longer gestation periods, and fewer nipples [22]. However, compared to their closest relatives in south-east Asia and the ancestral state of their common ancestor (Node B), the old endemics have similar breeding parameters. Thus, the slow reproductive rates of the Australian old endemics reflect retention of ancestral states and not adaptation to the unique Australian environment. The new endemics, in contrast, showed a phylogenetic trend towards increasing reproductive rates compared to the ancestors of the Murinae. In addition, the three species of the *sordidus* group were all nested phylogenetically within the new endemics, with reproductive rates that were significantly higher than the other new endemics. Thus, within the new endemics there has been an evolutionary trend towards increased reproductive rates as they expanded throughout Australia, particularly into the climatically erratic regions of the interior, the monsoonal north, and the tropical grasslands of the north-east. Similarly, the house mouse, which is better adapted to arid conditions, has invaded almost the entire continent, while the two rat species are restricted to coastal areas, mainly near human habitations. The already elevated reproductive rate of the house mouse has perhaps aided it to invade the open grasslands and arid habitats, where it behaves in a similar way to that of the long-haired rat and other irruptive species. While the slow reproductive rates of the old endemics may be compatible with the Australian environment, the high reproductive rates of the new endemics suggest that these are an equally adaptive solution to the same environment.

Our analyses of among-colonization stages suggest that overall there is no correlation between breeding parameters and climate within each group. Indeed, the old endemics have almost no variation in litter size (SD = 0.68 compared with 2.15 in the new endemics and 1.25 in the introduced species) and no variation in nipple number. This suggests that these breeding parameters lack the variation to respond to selection and are constrained by their phylogenetic ancestry. Despite their much more recent colonization and common ancestry, the new endemics, in contrast, have significantly more variation in both nipple number and litter size, which vary even within species. We conclude that the lack of correlation among Australian rodents between breeding parameters and these climatic variables implies that eco-region/habitat type is not a key factor in explaining variation in breeding performance. This conclusion does not mean that climate is not a strong selective force within species, but that we lack the data to test the effect of climate on variation within species.

A third hypothesis for the difference in reproductive rates between the old endemics and new endemics is that each of these groups competes with the other and they have consequently forced to exploit different resources. Several field studies and removal experiments in habitats where representatives of both the old endemic rodents and the new endemics live sympatrically provide evidence for competition between the colonization stages [45]–[46]. A series of studies on intraspecific competition between the old endemics *Pseudomys gracilicaudatus/higginsi* and the new endemic, *Rattus lutreolus*, showed that: 1) these species partition one or more resources in order to coexist [46]; 2) *Pseudomys* is more opportunistic in diet than *R. lutreolus* [47]; 3) the larger *Rattus* is behaviorally dominant over *Pseudomys* [48]; and 4) there is a dynamic balance of stable competitive coexistence between these species based on various stages of post-fire plant succession [49]. Most importantly, these studies showed that there is a strong competitive interaction between the two groups. While they suggested that the old endemics occupy inferior microhabitats when living sympatrically with the new endemics [50], each of these studies examined interactions between a larger new endemic and a smaller old endemic species. Our own data have shown that, collectively, the old endemics do not differ in mean body mass from the new endemics. However, while the latter range in body mass from about 70 to 150 g, the former have a much broader range, from about 10 g (*Pseudomys delicatulus*) to over 500 g (*Uromys* and *Hydromys*). No studies have considered the competitive interactions between the several large species of old endemics and the new endemics (but see [51]–[52] for other studies of competition between some Australian rodents). Although the species of the different colonization stages compete for limited resources, they have long co-existed in the same habitats. We suggest therefore that the two reproductive modes displayed by the old and new endemics, respectively, constitute successful alternative strategies.

In conclusion, we have shown that the reproductive rates of the Australian rodents can be predicted primarily from their colonization stage, similar to the Australian passerines [20], [42]. Clearly, colonization stages are largely defined by phylogeny, particularly for the two native radiations, and phylogeny alone is a strong predictor of breeding variables. In addition, because climatic variables revealed no significant correlation with either reproductive rate or colonization stage, it would appear that rodents of the different colonization stages have responded to the same environments with differential reproductive adaptations. Despite their ecological and morphological disparity, the old endemic rodents have retained to a great extent the low reproductive rates of their ancestors in tropical south-east Asia. In contrast, the new endemics, with their significantly higher ancestral reproductive rates, show a clear trend of increasing fecundity as they diversified in an Australia already saturated with rodents. Both strategies represent successful alternatives for survival in the unique environmental conditions of Australia.

## Supporting Information

Table S1 Breeding parameters for the Australian rodents. Extinct species are marked with an asterisk. Data were compiled from the following sources: [18], [22], [26], [27], [28].
(DOC)
