# Supplementary material for: Reproductive Rates in Australian Rodents Are Related to Phylogeny
Source: PLoS One. 2011 Apr 29;6(4):e19199. doi: 10.1371/journal.pone.0019199 (PMC3084793; doi:10.1371/journal.pone.0019199)
Supplement: Table S1 — Breeding parameters for the Australian rodents. Extinct species are marked with an asterisk. Data were compiled from the following sources: [18], [22], [26], [27], [28]. (DOC) [file pone.0019199.s001.doc]

Table S1: Breeding parameters for the Australian rodents. Extinct species are marked with an asterisk. Data were compiled from the following sources: [18, 22, 26, 27, 28].

| Species | Weight | Litter size | No. of nipples | Gestation | Weaning | Sexual maturity |
| --- | --- | --- | --- | --- | --- | --- |
|  | (g) |  |  | (days) | (days) | (months) |
|  |  |  |  |  |  |  |
| *Conilurus albipes** | 200 | 2.5 | 4 |  | 20.0 |  |
| *Conilurus penicillatus* | 150 | 2.0 | 4 | 36.0 | 20.0 | 3.0 |
| *Hydromys chrysogaster* | 685 | 4.0 | 4 | 34.0 | 32.0 | 8.0 |
| *Leggadina forresti* | 20 | 3.5 | 4 | 35.0 | 28.0 |  |
| *Leggadina lakedownensis* | 17 | 3.0 | 4 | 30.0 |  | 3.0 |
| *Leporillus apicalis** | 150 |  | 4 |  |  |  |
| *Leporillus conditor* | 350 | 2.0 | 4 | 44.0 | 30.0 | 8.0 |
| *Mastacomys fuscus* | 122 | 2.5 | 4 | 39.0 | 35.0 | 10.0 |
| *Melomys burtoni* | 55 | 2.5 | 4 |  | 21.0 |  |
| *Melomys capensis* | 80 | 2.0 | 4 |  |  | 3.0 |
| *Melomys cervinipes* | 80 | 2.0 | 4 | 38.0 | 20.0 | 5.0 |
| *Melomys rubicola* | 100 |  | 4 |  |  |  |
| *Mesembriomys gouldi* | 580 | 2.0 | 4 | 43.0 | 42.0 | 3.0 |
| *Mesembriomys macrurus* | 267 | 2.0 | 4 | 47.0 | 45.0 | 10.0 |
| *Mus musculus* | 17 | 6.0 | 10 | 19.0 | 18.0 | 2.0 |
| *Notomys alexis* | 35 | 3.5 | 4 | 32.0 | 30.0 | 3.0 |
| *Notomys aquilo* | 39 | 3.0 | 4 | 49.0 |  |  |
| *Notomys cervinus* | 35 | 3.0 | 4 | 40.0 | 28.0 | 6.0 |
| *Notomys fuscus* | 35 | 3.0 | 4 | 34.0 | 29.0 | 3.0 |
| *Notomys longicaudatus** | 100 |  | 4 |  |  |  |
| *Notomys macrotis** | 50 |  | 4 |  |  |  |
| *Notomys mitchelli* | 52 | 3.5 | 4 | 32.0 | 35.0 | 3.0 |
| *Pogonomys mollipilosus* | 62 | 2.5 | 6 |  |  |  |
| *Pseudomys albocinereus* | 26 | 4.0 | 4 | 38.0 |  | 2.5 |
| *Pseudomys apodemoides* | 20 | 4.0 | 4 | 38.0 | 40.0 | 15.0 |
| *Pseudomys australis* | 65 | 4.0 | 4 | 30.0 | 28.0 | 2.8 |
| *Pseudomys calabyi* | 17 | 3.0 | 4 |  |  |  |
| *Pseudomys chapmani* | 10 | 4.0 | 4 |  |  |  |
| *Pseudomys delicatulus* | 10 | 3.0 | 4 | 31.0 | 30.0 |  |
| *Pseudomys desertor* | 25 | 3.5 | 4 | 28.0 | 20.0 | 2.5 |
| *Pseudomys fumeus* | 70 | 3.5 | 4 |  |  |  |
| *Pseudomys gouldi** | 50 |  | 4 |  |  |  |
| *Pseudomys gracilicaudatus* | 63 | 3.0 | 4 | 27.0 | 28.0 |  |
| *Pseudomys hermannsburgensis* | 12 | 3.5 | 4 | 31.0 | 30.0 | 3.0 |
| *Pseudomys higginsi* | 67 | 3.5 | 4 | 31.0 | 25.0 |  |
| *Pseudomys johnsoni* | 12 |  | 4 |  |  |  |
| *Pseudomys laborifex* | 12 | 2.5 | 4 |  |  |  |
| *Pseudomys nanus* | 34 | 3.0 | 4 | 24.0 | 18.0 | 2.5 |
| *Pseudomys novaehollandiae* | 16 | 3.5 | 4 | 33.0 | 25.0 | 3.0 |
| *Pseudomys occidentalis* | 34 | 3.5 | 4 |  |  |  |
| *Pseudomys oralis* | 90 | 2.5 | 4 |  |  |  |
| *Pseudomys pilligaensis* | 11 | 3.0 | 4 | 27.0 |  |  |
| *Pseudomys praeconis (fieldi)* | 45 | 3.5 | 4 | 24.0 | 30.0 |  |
| *Pseudomys shortridgei* | 70 | 3.0 | 4 |  |  | 10.5 |
| *Rattus colletti* | 61 | 9.0 | 12 | 22.5 | 20.0 | 1.2 |
| *Rattus exulans* | 80 | 3.5 | 8 | 22.0 | 20.0 | 2.5 |
| *Rattus fuscipes* | 125 | 5.0 | 10 | 23.0 | 31.0 | 4.0 |
| *Rattus leucopus* | 116 | 3.5 | 6 | 23.0 | 27.5 | 3.0 |
| *Rattus lutreolus* | 122 | 5.0 | 10 | 22.0 | 21.0 | 3.0 |
| *Rattus norvegicus* | 320 | 8.5 | 12 | 22.0 | 20.0 | 1.5 |
| *Rattus rattus* | 280 | 7.5 | 12 | 22.0 | 20.0 | 3.5 |
| *Rattus sordidus* | 125 | 7.5 | 12 | 22.0 | 20.0 | 2.5 |
| *Rattus tunneyi* | 76 | 4.5 | 10 | 22.0 | 21.0 | 1.2 |
| *Rattus villosissimus* | 112 | 8.5 | 12 | 22.5 | 21.0 | 2.3 |
| *Uromys caudimaculatus* | 545 | 2.0 | 4 | 36.0 |  | 6.0 |
| *Uromys hadrourous* | 179 |  | 4 |  |  |  |
| *Xeromys myoides* | 41 |  | 4 |  |  |  |
| *Zyzomys argurus* | 45 | 2.5 | 4 | 34.0 | 28.0 | 5.5 |
| *Zyzomys maini* | 94 | 2.5 | 4 | 35.0 | 28.0 | 5.5 |
| *Zyzomys palatalis* | 123 |  | 4 |  |  |  |
| *Zyzomys pendunculatus* | 70 |  | 4 |  |  |  |
| *Zyzomys woodwardi* | 130 | 1.5 | 4 |  | 28.0 | 5.5 |
